# Quantifying size-dependent interactions between fluorescently labeled polystyrene nanoparticles and mammalian cells

**DOI:** 10.1186/1477-3155-10-39

**Published:** 2012-09-24

**Authors:** Juan A Varela, Mariana G Bexiga, Christoffer Åberg, Jeremy C Simpson, Kenneth A Dawson

**Affiliations:** 1 Centre for BioNano Interactions, School of Chemistry and Chemical Biology, University College Dublin, Belfield, Dublin, 4, Ireland; 2 School of Biology and Environmental Science and Conway Institute of Biomolecular and Biomedical Research, University College Dublin, Belfield, Dublin, 4, Ireland; 3 PhD Programme in Experimental Biology and Biomedicine, Centre for Neurosciences and Cell Biology, University of Coimbra, 3004-517, Coimbra, Portugal

**Keywords:** Bio-nanotechnology, Cellular uptake, Nanoparticles, Single particle tracking

## Abstract

**Background:**

Nanoparticles (NPs) are currently used in a wide variety of fields such as technology, medicine and industry. Due to the novelty of these applications and to ensure their success, a precise characterization of the interactions between NPs and cells is essential.

**Findings:**

The current study explores the uptake of polystyrene NPs by 1321N1 human astrocytoma and A549 human lung carcinoma cell lines. In this work we show for the first time a comparison of the uptake rates of fluorescently labeled carboxylated polystyrene (PS) NPs of different sizes (20, 40 and 100 nm) in two different cell types, keeping the number of NPs per unit volume constant for all sizes. We propose a reliable methodology to control the dose of fluorescently labeled NPs, by counting individual NPs using automated particle detection from 3D confocal microscopy images. The possibility of detecting individual NPs also allowed us to calculate the size of each nanoparticle and compare the fluorescence of single NPs across different sizes, thereby providing a robust platform for normalization of NP internalization experiments as measured by flow cytometry.

**Conclusions:**

Our findings show that 40 nm NPs are internalized faster than 20 nm or 100 nm particles in both cell lines studied, suggesting that there is a privileged size gap in which the internalization of NPs is higher.

## Background

Nanoparticles (NPs) can be defined as ultra fine particles with lengths between 1 nm to 100 nm in at least two of their dimensions. Currently nanomaterials are being used in a wide variety of applications such as engineering, food industry, cosmetics and medicine [1,2]. In medicine there are major expectations for the use of nanoparticles to facilitate targeted drug delivery [3-5]. Due to the novelty of these applications and to ensure their success, a precise characterization of the interactions between NPs and cells is essential.

Polystyrene (PS) NPs are widely used as a model to study interactions between NPs and cells due to various practical reasons including their commercial availability, high quality and wide variety of size and surface chemistry. These NPs have been reported to enter different cell types including hepatocytes [6], macrophages [7] and lung [8]. One general conclusion is that particles smaller than 100 nm are able to enter mammalian cells. The specific uptake pathways of these NPs, as well as the uptake rates, have been shown to be cell type- [9], NP size- [10,11] and shape-dependent [12] but are also related to the surface chemistry of the NP [13] and its hydrophobicity [14]. Although various studies indicate that the final localization of NPs is usually the lysosome [7,15,16], the internalization mechanism is not fully understood [17]. Indeed, multiple mechanisms may intervene in parallel as we previously observed for both A549 and 1321N1 cells [18].

At present, most studies comparing the effects of the uptake of different sized NPs are based on exposure to the same concentration of particles measured in mass per unit volume. Using this approach, studies on the cellular uptake of different sized NPs [19,20], as well as on their toxicity [6,21], have shown size-dependent effects. Although this kind of particle exposure can provide useful information, it also presents clear drawbacks in the understanding of the interactions between cells and NPs, specifically in distinguishing whether the reported effects were due to the size or simply to a difference in NP number (e.g. there are two orders of magnitude between the actual number of 20 nm and 100 nm NPs when used at the same mass per unit volume concentration). These large differences in NP numbers may bias results when investigating toxicity, internalization, and intracellular traffic of different sized NPs, as these interactions are dose-dependent.

## Results and discussion

In this study we used fluorescently labeled carboxylated (−COOH) PS NPs (measuring 20, 40 and 100 nm in diameter) to understand the effects of size in their uptake by 1321N1 and A549 cells. We began by carefully characterizing the NPs in phosphate buffered saline (PBS) at pH 7.0 and 25°C as described in the Additional file 1: Experimental Section. The hydrodynamic size of the NPs in PBS was measured by dynamic light scattering (DLS), revealing that they were well dispersed in all cases (Table 1), as all measurements presented a low polydispersity index. The ζ-potential of the NPs was also determined and as expected from the chemistry of the NPs, all displayed a negative ζ-potential (Table 1), indicating that these NPs present a negative surface charge. NPs were also characterized in complete cell culture medium (Additional file 1: Table S1) revealing that they continued to be monodispersed, although the measured size was larger than that in PBS, most likely a consequence of the protein corona surrounding the NPs [22]. The ζ-potential of the particles in cell culture medium was approximately zero, suggesting a screening effect of the NP surface charge due to the proteins present in the medium.

**Table 1:** Dynamic light scattering characterization of PS NPs used

**NP Denomination**	**Hydrodynamic Size [nm]**	**PDI [a]**	**ζ-potential [mV]**
20 nm	33 ± 1	0.14	−27 ± 3
40 nm	44 ± 1	0.13	−24 ± 3
100 nm	114 ± 4	0.01	−34 ± 2

All particles were characterized in PBS at pH 7.0 and 25°C.

[a] Polydispersity Index.

In order to expose cells to a fixed number of NPs we first estimated the number of NPs present in 1 mL of solution, at a certain concentration *C* in g/mL. If the density of the particles is *ρ* (in g/mL) and the diameter *d* (in μm), an estimation of the number of NPs/mL would be given by:

(1) $$\frac{\text{Number of particles}}{mL}=\frac{6C\times 10^{12}}{\pi d^{3}\rho }$$

**Figure 1:**
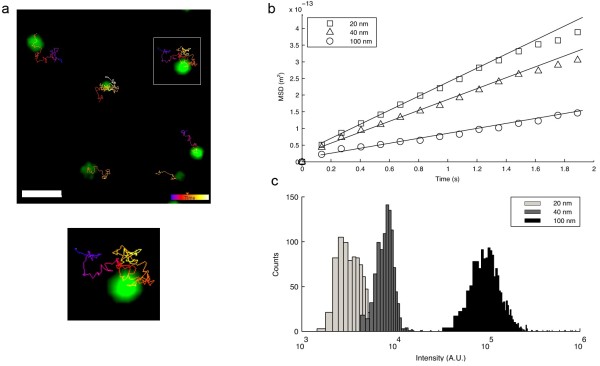
**Assessment of individual nanoparticle size and fluorescence.** Automated single NP tracking was performed using a spinning disk confocal microscope. **a.** Green fluorescent 100 nm NPs were dispersed in glycerol at 40°C and their trajectories imaged and analyzed. NPs are identified as a green spot and the trajectory is displayed by a multicolored line (scale bar corresponds to 3 μm). The NP trajectory enclosed in the white square is shown enlarged below. **b.** The diffusion coefficients and NP sizes were obtained from the slope of the plots of MSD versus time (linear fits are shown by continuous lines). **c.** The fluorescence of individual NPs was obtained from the confocal images in order to normalize experiments of NP uptake by cells, using the means of the populations shown in the histograms. A.U. – arbitrary fluorescence units.

However, the number of NPs/mL calculated in this way is only an approximation, as it relies on assumptions of both the stock concentration and the homogeneous particle size (see Additional file 1: Additional Information). In order to obtain a more precise measurement of the number of NPs present in solution we first established whether individual NPs could be identified from microscopy images. We therefore dispersed the NPs in 100% glycerol and imaged the NPs freely diffusing at 40°C in a spinning-disk confocal microscope. The high viscosity of glycerol (433 times more viscous than water at 40°C) made NPs diffuse sufficiently slow such that it enabled reconstruction of reliable trajectories, even of the 20 nm NPs. Images were then processed with Imaris software by performing automated particle detection and subsequent tracking routines (Figure 1a). There are several other available software packages to track particles. We chose Imaris due to its good performance and three dimensional (3D) rendering capabilities, allowing easy visual inspection of the detection and tracking accuracy. Larger NPs (100 nm) could be tracked for a high number of frames without detectable bleaching (over 200 frames for 100 nm NPs), whereas 20 nm particles typically started to bleach after 50 to 100 frames. From the trajectories obtained, the mean square displacement (MSD) was calculated (see Additional file 1: Additional Information), and the diffusion coefficient derived from the plots of MSD versus time, as freely diffusing particles present a linear relation between the MSD and time. The Stokes-Einstein relation (Equation 2) applied to the diffusing particle was then used to obtain the radius of the particle (*a*):

(2) $$D=\frac{k_{B}T}{6\pi \eta a}$$

where *k*_B_ is Boltzmann's constant, *T* is the temperature in Kelvin and *η* the fluid (dynamic) viscosity. The MSD versus time plots for 20 nm, 40 nm and 100 nm PS-COOH NPs freely diffusing in glycerol (Figure 1b) allowed us to calculate the diffusion coefficients by fitting the data with first order polynomials. The particle size was then calculated using Equation 2. The sizes determined from the MSD analyses (Table 2) were very similar to those obtained by DLS (Table 1), thereby confirming that we were indeed observing individual NPs.

**Table 2:** Calculated numbers of PS NPs in stocks versus automatic count obtained from 3D confocal microscopy

**NP Denomination**	**SPT [a] calculated size [nm]**	**Estimated NP number [NP/mL]**	**Experimental NP count [NP/mL]**	**SPT Fluorescence Intensity [A.U.]**
20 nm	32	4.5 × 10^15^	2.2 × 10^15^	1.0
40 nm	42	1.5 × 10^15^	5.7 × 10^14^	2.3
100 nm	92	3.6 × 10^13^	3.7 × 10^13^	31.0

[a] Single particle tracking.

This allowed us to determine the absolute concentration of the NP stock in number of NPs per unit volume (Table 2). For this, a NP count was performed in glycerol from 3D images, each consisting of 50 confocal slices obtained with a spinning disk confocal microscope. After counting over 20,000 NPs for each case, and taking into account the volume of the z-stack obtained with the microscope, the number of NPs per unit volume in the dispersion, and therefore stock, was calculated (Table 2). The mean fluorescence of the detected NPs was also calculated (Figure 1c and Table 2) and this result was used to normalize subsequent experiments carried out in cells, by dividing flow cytometry fluorescence measurements by the mean fluorescence yield of each NP size. This normalization step takes into consideration that larger NPs are brighter than smaller ones. The number of detected NPs per unit volume was then compared with the estimated number of NPs per unit volume (using Equation 1) and we concluded that the values differed one fold for the 20 and 40 nm NPs, while for 100 nm NPs the experimental count coincided with the estimated number (Table 2). This is of extreme importance for biological experiments where it is necessary to use the same number of particles if accurate comparisons of phenotypic effects are to be made.

**Figure 2:**
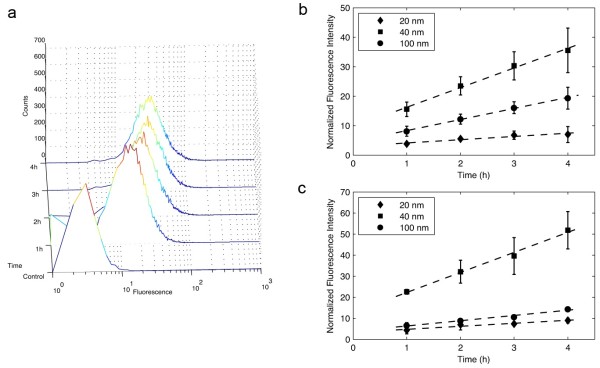
**Nanoparticle uptake kinetics in 1321N1 and A549 cells.** Cells were incubated with NPs of different sizes maintaining the number of particles constant at 6x10^11^ particles/mL. **a.** The full cell population behavior is shown for 1321N1 cells incubated with 40 nm NPs without normalization. As the populations displayed a single peak, the population mean was used for comparisons across the different NP sizes. The kinetics of NP uptake for the three NP sizes is shown for 1321N1 (**b**) and A549 cells (**c**). Plotted data points correspond to the average of three independent experiments, each with two replicates. Dashed lines correspond to linear fits. Error bars correspond to standard deviations between the three independent experiments.

In order to quantify and study the kinetics of PS-COOH NP uptake, cells were incubated with the different sized NPs for increasing lengths of time (1, 2, 3 and 4 h) and the cell-associated fluorescence measured by flow cytometry. The arithmetic mean of the cell populations was compared across different samples, as the histograms of fluorescence intensity presented clear single peaks (Figure 2a, Additional file 1: Figure S1). Cells were exposed to 6 × 10^11^ NPs/mL in complete cell culture medium for each NP type used. Analysis of the uptake kinetics for both cell lines showed that after a first transient non-linear regime (the control fluorescence corresponds to 0 value in the plots), the internalization of NPs was proportional to the incubation time of the experiment. To determine the rate of internalization of the NPs, we performed linear fits by least squares calculation for the interval between 1 and 4 h and determined the slope of the line, which corresponded to the rate of uptake. In order to compare across the different scenarios, all uptake rates were normalized to the uptake rate of the 20 nm nanoparticles in 1321N1 cells. The uptake rate for 40 nm was 6.7 and for 100 nm NPs 3.8 (in arbitrary fluorescence units (A.U.) per hour, Figure 2b). For A549 cells the rates of uptake of the NPs followed a similar trend, with the 20 nm NPs being those with the slowest uptake rate (1.4 A.U./h) and 40 nm NPs those that entered cells the most rapidly (9.5 A.U./h, Figure 2c). The uptake rate obtained for the 100 nm NPs (2.5 A.U/h) was slower than for 1321N1 cells. As the cells were exposed to the same number of NPs, and the rates of uptake were different, these results may suggest that the mechanisms by which NPs of 40 nm or 100 nm are imported by the cell differ. It should be noted that the adhesion of larger NPs to the cell surface could be stronger than that of smaller NPs; e.g. the van der Waals force between a sphere and a surface is proportional to the diameter of the sphere [23]. Despite this, the uptake of 40 nm particles was faster than that of 100 nm particles, which might be explained by different kinetics of the endocytosis mechanisms utilized by the cells to internalize the NPs. In the case of the 20 nm NPs, van der Waals adhesion forces are smaller and the diffusion higher, which could contribute to the lower uptake rate when compared with the two larger NPs studied here.

In order to confirm that the PS-COOH NPs were being internalized by 1321N1 and A549 cells and to investigate the intracellular trafficking of the NPs, we performed immunofluorescence for EEA1 and LAMP1, early endosomal and lysosomal markers, respectively, followed by confocal microscopy imaging. We studied the localization of the nanoparticles after 2 h of incubation with the same NP exposure conditions as for the flow cytometry experiments. These experiments revealed co-localization of NPs with both markers, suggesting that NPs follow the endosomal-lysosomal pathway inside cells (Additional file 1: Figures S2 and S3).

## Conclusions

Our correlation between single particle tracking (SPT) analysis of NPs in solution and flow cytometry experiments measuring NP internalization proved to be a robust method to investigate bio-nano interactions. We observed that the number of NPs present in a particle stock may differ from the estimated numbers, and therefore careful characterization of the nanomaterials (including measuring the actual number of NPs) is important to quantitatively assess effects in biological environments. NP uptake rates were shown to differ between the two cell lines under study (Additional file 1: Figure S4) with the uptake rates being NP-size-dependent for each cell line. For both cell lines under study 40 nm NPs were internalized faster than 20 nm or 100 nm NPs, consistent with previous literature on gold nanoparticles [24]. This could suggest different internalization mechanisms for the different NP sizes, although this will need further clarification.

The ability to characterize NP dispersions at a single particle level provided us with the advantage of being able to obtain size-dependent NP uptake rates, that would have been meaningless if the cells had been exposed to different NP numbers. One clear strength of the presented method is that the NP dispersion is characterized in terms of size and fluorescence through SPT, yielding valuable information for the interpretation of results. The method is sufficiently simple that it can be routinely implemented prior to biological experiments investigating interactions between NPs and cells. One drawback, however, is that the fluorescently labeled NPs need to be sufficiently bright to be able to detect them individually. In our opinion, the standardization methodology that we describe here is appropriate for taking account of size effects of different NPs, and as such can be applied to a variety of experiments such as quantification of NP internalization, toxicity and trafficking. Furthermore, alternative experimental strategies such as keeping the total NP surface constant across different NP sizes could also be applied using this methodology. This work therefore provides a methodology that should be applicable to many future studies aiming to derive a greater and more quantitative understanding of the interactions between NPs and biological systems.

## Abbreviations

3D: 3 dimensional; A.U.: Arbitrary fluorescence units; DLS: Dynamic light scattering; MSD: Mean square displacement; NPs: Nanoparticles; PBS: Phosphate buffered saline; PS: Polystyrene; SPT: Single particle tracking.

## Competing interests

The authors declare that they have no competing interests.

## Authors’ contributions

JAV conceived, designed, performed and analyzed experiments and wrote the manuscript. MGB performed experiments and wrote the manuscript. CÅ designed and analyzed experiments. JCS wrote the manuscript. KAD supervised and coordinated the project. All authors read and approved the manuscript.

## Supplementary Material

Additional file 1 Additional Information. Click here for file
