# Supplementary material for: Quantifying size-dependent interactions between fluorescently labeled polystyrene nanoparticles and mammalian cells
Source: J Nanobiotechnology. 2012 Sep 24;10:39. doi: 10.1186/1477-3155-10-39 (PMC3492040; doi:10.1186/1477-3155-10-39)
Supplement: Additional file 1 — Additional Information. [file 1477-3155-10-39-S1.pdf]

Additional Information

Quantifying size-dependent interactions between fluorescently labeled polystyrene nanoparticles and mammalian cells

Juan A. Varela, Mariana G. Bexiga, Christoffer Åberg, Jeremy C. Simpson and Kenneth A. Dawson

Experimental Section

Cell culture. Human brain astrocytoma 1321N1 and human lung carcinoma A549 cells were cultured at 37 °C in 5% CO_2 in Minimal Essential Medium supplemented with 10% fetal bovine serum (Gibco) and 1% non-essential amino acids (HyClone). Cells were typically sub-cultured 1:3 every second day by incubating them in 0.25% trypsin (Gibco).
Regular mycoplasma tests were carried out, using the mycoAlert kit (Lonza Inc.).

Nanoparticle characterization. Yellow-green carboxylated polystyrene NPs (FluoSpheres) with mean diameters of 0.02 μm, 0.04 μm, and 0.1 μm were purchased from Molecular Probes and were used without further chemical modification. Size and ζ-potential were determined using a Malvern Zetasizer Nano Series. Polystyrene NPs were diluted to 50 μg/mL or 250 μg/mL in PBS or complete growth medium before measurement. Measurements were conducted at pH 7.0 and 25 °C.

Spinning disk confocal microscopy of NPs. Single color visualization of NPs in solution was performed on a spinning disk confocal microscopy system consisting of a CSU22 spinning disk unit (Yokogawa Electric corporation) and an iXon+ 897 EMCCD camera (Andor), mounted on a IX81 inverted microscope (Olympus) equipped with a climate control chamber. To perform single particle tracking analysis and to measure the NP concentration, yellow-green fluorescent NPs were diluted in 100% glycerol at 40 °C and were excited with a 488 nm laser line. A 60x 1.35 NA

Olympus UPlanSAPO oil immersion objective was used to visualize the samples. Two dimensional images were acquired at approximately 100 frames per second, while 3D images for NP counts were acquired at approximately 1 stack per second (consisting of 50 slices). Images were acquired using Andor iQ software (Andor Technology) and analyzed with Imaris software (Bitplane).

NP uptake measurements by flow cytometry. Cells growing in 12-well plates ($2x10^4$ cells/cm^2) were incubated with yellow-green fluorescently labeled PS-COOH NPs of 20 nm, 40 nm or 100 nm, keeping the number of particles constant at approximately $6x10^{11}$ particles per mL. NP dispersions were prepared by directly diluting NPs from the stocks in complete growth medium at room temperature. The cell growth medium was then replaced with medium containing NPs and cells were returned to the incubator. At each time point (1 h, 2 h, 3 h, 4 h), cells incubated with the different NP sizes (3 replicates for each size and negative controls without NPs) were removed from the incubator, then washed, fixed and harvested as followed. Cells were washed 3 times with 1 mL PBS, harvested with 0.25% trypsin (Gibco) for 4 min at 37 °C and fixed in 3% paraformaldehyde (PFA) for 20 min at room temperature. The PBS washes and subsequent trypsin treatment were efficient in detaching NPs adhering to the surface of the cell. Analysis of fluorescence was performed using a Cyan ADP cytometer (DAKO) exciting at 488 nm and measuring fluorescence emission at 530 nm. Gating was performed to exclude events that had very low forward scattering and size scattering. Post-acquisition analysis was carried out using Matlab software (Mathworks) and Summit software (DAKO). Flow cytometry was carried out in the Flow Cytometry Core Facility of the Conway Institute for Biomolecular and Biomedical Research, University College Dublin.

Immunostaining. Cells were seeded on sterile coverslips in 12-well plates. For 1321N1 cells, the coverslips had been previously coated with rat tail collagen type I. After 24 h, cells were treated with yellow-green fluorescently labeled PS-COOH NPs of 20 nm, 40 nm or 100 nm, keeping the number of particles constant at approximately $6x10^{11}$ particles per mL as described for the Flow Cytometry experiments. After 2 h of incubation, NPs were removed and cells were washed 3 times with 1 mL PBS. Cells were then fixed with 3% PFA for 20 min at room

temperature followed by permeabilization with 0.1% Triton-X100 for 5 min at room temperature. Once permeabilized, cells were incubated with mouse anti-human EEA1 antibody (BD, clone 14/EEA1) diluted 1:200 in PBS or mouse anti-human LAMP1 antibody (DHSB, clone H4A3-a) diluted 1:200 in PBS, for 1 h at room temperature. Coverslips were then washed and incubated for 30 min at room temperature with phalloidin-Alexa Fluor 647 (Molecular Probes) diluted 1:40 in PBS, together with the secondary anti-mouse antibody conjugated to Alexa Fluor 568 (Molecular Probes) diluted 1:200 in PBS. Coverslips were mounted on slides using Mowiol (Sigma). Slides were visualized with a point scanning confocal microscope (Olympus FV1000) using a 60x 1.35 NA Olympus UPlanSAPO oil immersion objective. Images were acquired in a sequential scanning mode. Three dimensional stacks of over 10 cells for each case were acquired.

Additional information for the estimation of nanoparticle number in dispersions:

We present here the method to estimate the number of NPs present in 1 mL of solution, at a certain concentration *C* in g/mL. For this, we assume that all particles are spheres of equal diameter - *d* - in cm, and so the number of particles can be obtained by dividing the total mass of particles by the mass of a single particle, which in terms of the density - ρ - of the particles (1.05 g/mL for polystyrene) would be:

$$\frac{\text{Number of particles}}{mL} = \frac{\text{Total mass of particles}/mL}{\text{mass of 1 particle}} = \frac{C}{\frac{4}{3}\pi(\frac{d}{2})^3\rho} = \frac{6C}{\pi d^3\rho}$$

The diameter of the particle is usually expressed by the NP manufacturer in μm:

$$\frac{\text{Numberof particles}}{mL} = \frac{6C\text{x}10^{12}}{\pi d^3\rho}$$

Additional information for single particle tracking analysis:

For a system of N particles, where $r_i(t)$-$r_i(0)$ is the vector distance traveled by a particle over the time interval t, the MSD can be defined as:

$$MSD = \left\langle r^2(t) \right\rangle = \frac{1}{N}\sum_{i=1}^{N}(r_i(t) - r_i(0))^2$$

The MSD of particles undergoing simple diffusion is given by:

$$\left\langle r^2(t) \right\rangle = 2dDt$$

where D is the diffusion coefficient, d is the dimensionality of the system, and t is the time. D can be obtained by fitting the curve MSD versus t to a linear equation. Then, the Stokes-Einstein relation applied to the diffusing particle can be used to obtain the radius of the particle (a):

$$D = \frac{k_B T}{6\pi\eta a}$$

where k_B is Boltzmann's constant, T is the temperature in Kelvin and η is the fluid (dynamic) viscosity.

Supplementary Table S1. *Dynamic light scattering characterization of PS NPs in complete growth medium.* All particles were characterized at pH 7.0 and 25 °C.

NP Denomination	Hydrodynamic Size [nm]	PDI [a]	ζ-potential [mV]
20 nm	62 ± 1	0.23	-4 ± 1
40 nm	84 ± 1	0.12	-11 ± 1
100 nm	257 ± 4	0.34	-8 ± 1

[a] Polydispersity Index

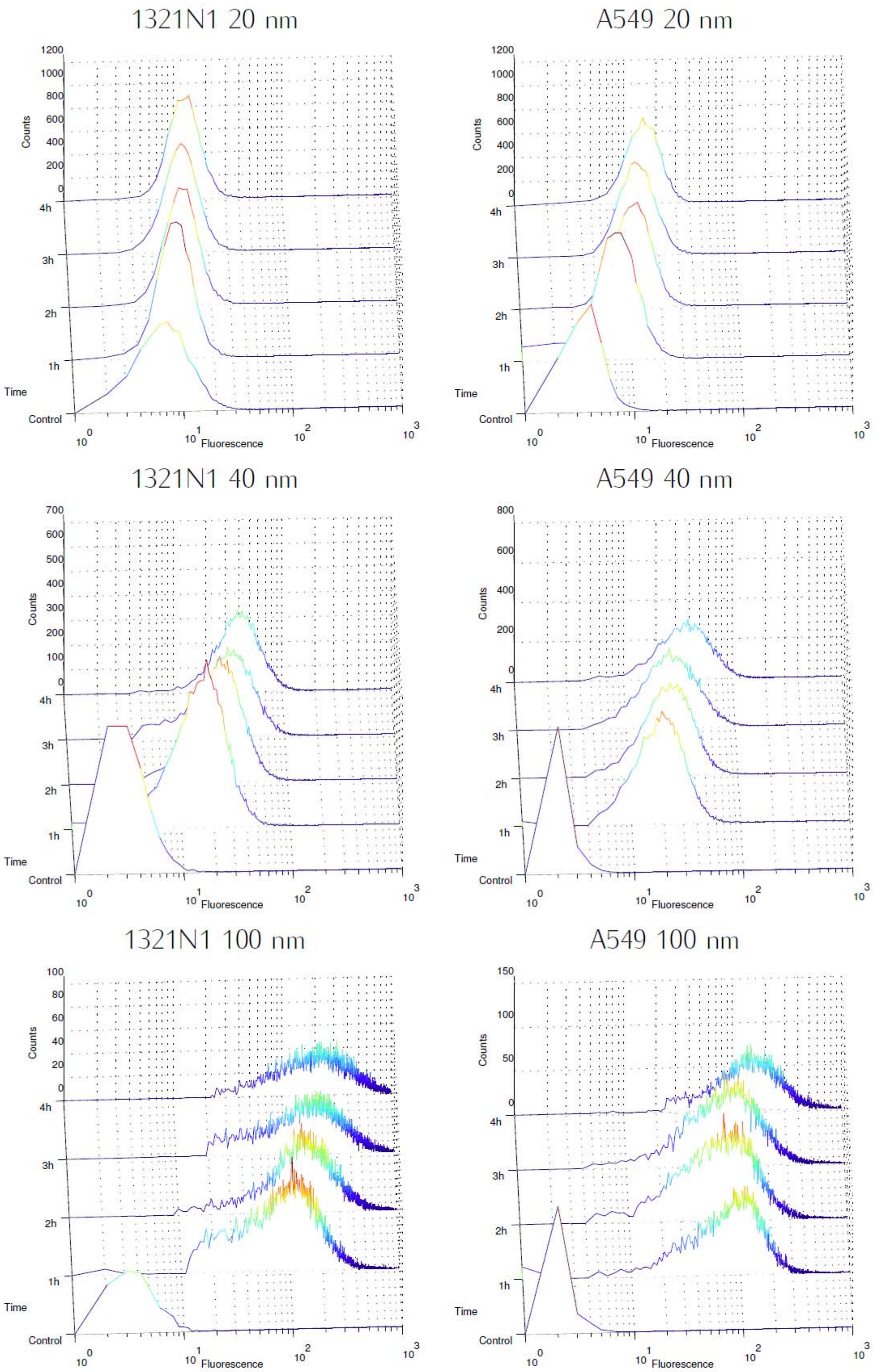

Supplementary Figure S1. *Nanoparticle uptake kinetics in A549 and 1321N1 cells.* Cells were incubated with NPs of different sizes maintaining the number of particles constant at $6x10^{11}$ particles/mL. The kinetics of NP uptake for 20 nm (upper), 40 nm (middle) and 100 nm (lower) NPs are shown for 1321N1 cells (left column) and A549 cells (right column). No normalization was performed.

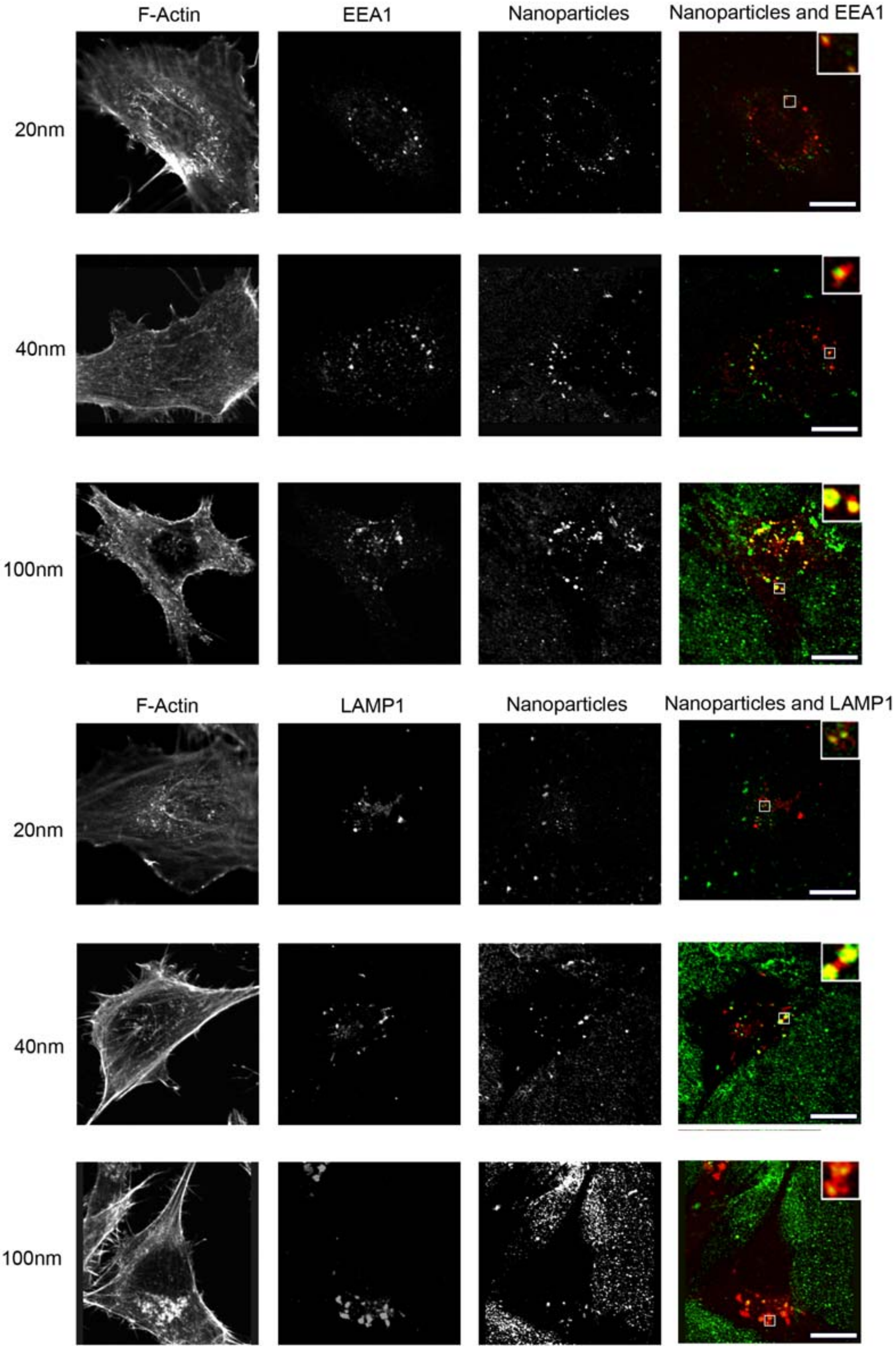

Supplementary Figure S2. *Confocal microscopy images of 1321N1 cells incubated with PS-COOH NPs of different sizes as indicated.* Confocal microscopy images of 1321N1 cells incubated with PS-COOH NPs of either 20 nm, 40 nm or 100 nm diameter for 2 h. NP number was kept constant for all different NPs used. In the merged images, NPs are shown in green and EEA1 (upper three rows) and LAMP1 (lower three rows) in red. Enlarged selections are shown in the upper right corner of merged images, and indicate co-localizing structures. Scale bars correspond to 20 μm.

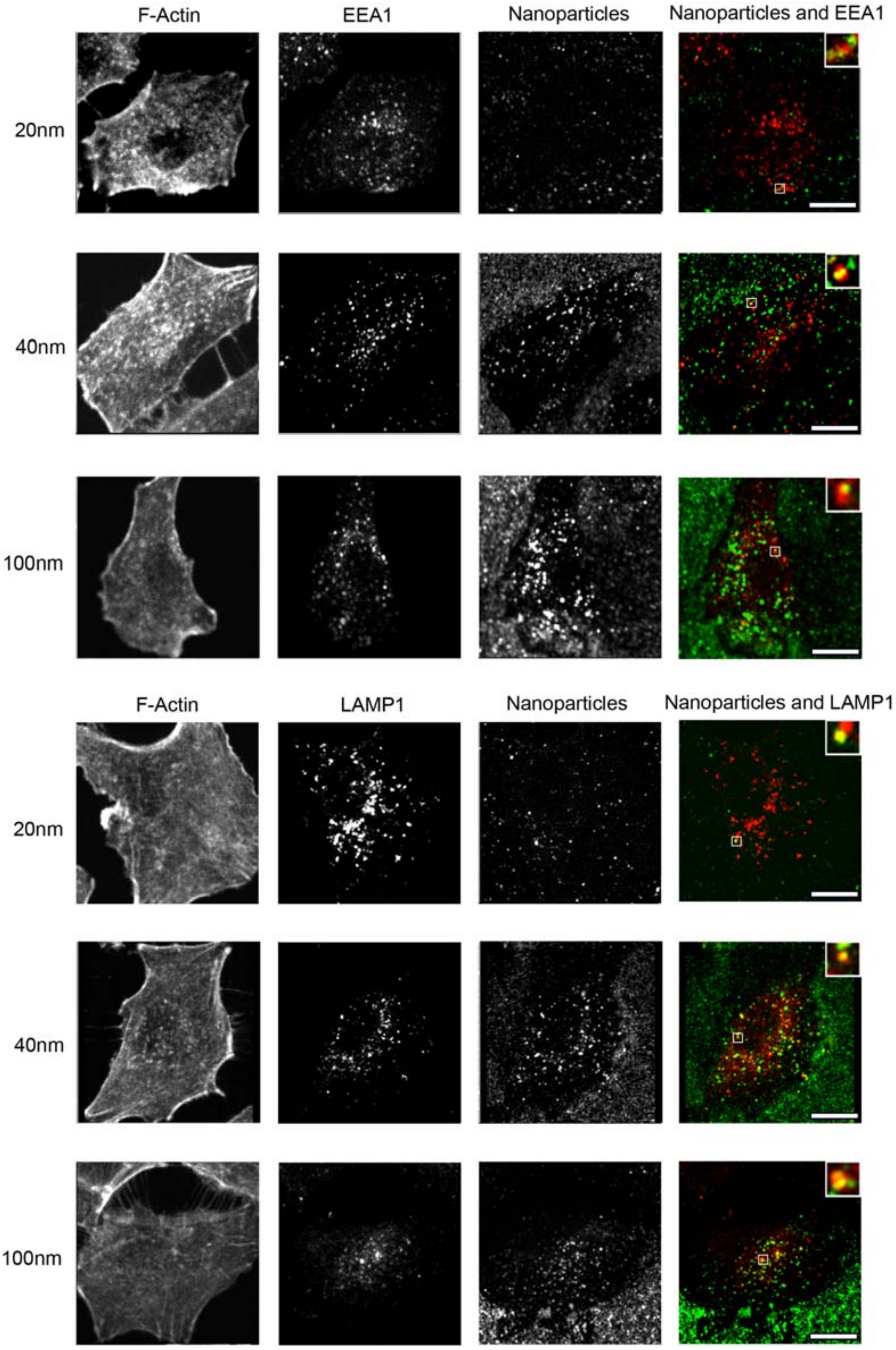

Supplementary Figure S3. *Confocal microscopy images of A549 cells incubated with PS-COOH NPs of different sizes as indicated.* Confocal microscopy images of A549 cells incubated with PS-COOH NPs of either 20 nm, 40 nm or 100 nm diameter for 2 h. NP number was kept constant for all different NPs used. In the merged images, NPs are shown in green and EEA1 (upper three rows) and LAMP1 (lower three rows) in red. Enlarged selections are shown in upper right corner of merged images showing co-localizing structures. Scale bars correspond to 20 μm.

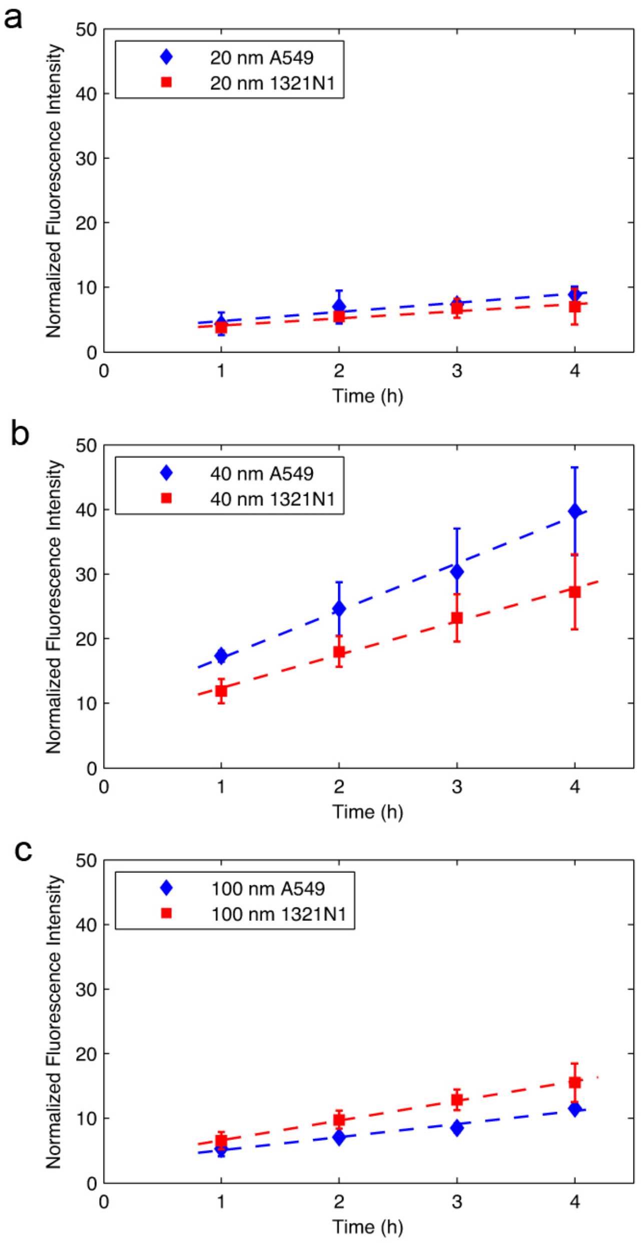

Supplementary Figure S4. *Nanoparticle uptake kinetics in 1321N1 and A549 cells.* Cells were incubated with NPs of different sizes maintaining the number of particles constant at $6x10^{11}$ particles/mL. The kinetics of NP uptake for 20 nm (**a**), 40 nm (**b**) and 100 nm (**c**) NPs is shown for 1321N1 and A549 cells. Normalization was carried out by subtracting the fluorescence means of the untreated cells to those of each sample and then dividing by the mean fluorescence of single NPs (Table 2 in the article). Plotted data points correspond to the average of three independent experiments, each with two replicates. Dashed lines correspond to linear fits. Error bars correspond to standard deviations between three independent experiments.
